# Women's abortion seeking behavior under restrictive abortion laws in Mexico

**DOI:** 10.1371/journal.pone.0226522

**Published:** 2019-12-27

**Authors:** Fatima Juarez, Akinrinola Bankole, Jose Luis Palma

**Affiliations:** 1 El Colegio de México, Tlalpan, Ciudad de México, Mexico; 2 Guttmacher Institute, New York, New York, United States of America; 3 Investigación Salud y Demografia, S. C., Tlalpan, Ciudad de México, Mexico; Queen's University at Kingston, CANADA

## Abstract

Abortion is regulated in Mexico at the state level, and it is permitted under certain criteria in all 32 states, except in Mexico City where first-trimester abortion is decriminalized. Yet, more than a million abortions occur in Mexico each year. But most terminations occurring outside of Mexico City are clandestine and unsafe due to profound stigma against the procedure, lack of trained providers, lack of knowledge of where to find a safe abortion and poor knowledge of the laws. While this situation is moderated by the increasing use of misoprostol, a relatively safe method of abortion, the safety of the procedure cannot be assured in restrictive legal contexts. The purpose of this study is to explore women’s experiences with induced abortion in three federal entities with different legal contexts, and whether abortion seeking behavior and experiences differ across these settings. The study was carried out in three states, representing three different degrees of restrictiveness of abortion legislation. Queretaro with the “most restrictive” law, Tabasco with a “moderately restrictive” law, and Mexico state with the “least restrictive” law. We hypothesize that women living in more restrictive states will resort to the use of more unsafe and risky methods and providers for their abortion than their counterparts in less restrictive states. Women who recently obtained abortions were selected through snowball sampling and qualitative data were collected from them using semi-structured indepth interviews. Data collection took place between mid-2014 and mid-2015, with a final sample size N = 60 (20 from each state). Various themes involved in the process of abortion seeking behavior were developed from the IDIs and examined here: women’s knowledge of the abortion law in their state, reasons for having an abortion; the methods and providers used and women’s positive and negative experiences with abortion methods and providers used. Our results indicate that abortion safety is not associated with the restrictiveness of abortion legislation. Findings show that there is a new pattern of abortion service provision in Mexico, with misoprostol, a relatively safe and easy to use method, playing an important role. Nevertheless, while access to misoprostol tends to increase the safety of abortion, the improvement is moderated by women and their informants (relatives, friends and partners) not having accurate information on how to safely self-induce an abortion with misoprostol. On the other hand, some women manage to have safe abortion in illegal setting by going to Mexico City or with the support of NGOs knowlegeable on abortion. Findings demonstrate the importance of decriminalization of abortion, but meanwhile, harm reduction strategies, including promotion of accurate information about self-use of misoprostol where abortion is legally restricted will result in safe abortion.

## I. Introduction

A large number of Mexican women resolve unintended pregnancies through induced abortions each year. An estimated 1,026,000 induced abortions were performed in 2009; these abortions occurred everywhere in the country and in all age groups, with a higher prevalence among young women [1]. In Mexico, abortion is regulated at the state level, and it is being permitted on the basis of some criteria in all 32 states [2], for example, it is allowed in all the states for rape. But of the 32 *entidades federativas* (or federative entities) in Mexico, Mexico City (previously named Federal District or DF)–is the only one that has decriminalized first-trimester abortion. Thus, almost all terminations that occur elsewhere in the country are practiced clandestinely [1,3,4], and the safety of these abortions cannot be assured. Women who seek these procedures often do so at the risk of their health and social standing.

Even though abortion is permitted under some circumstances in all states, very few women seek a legal abortion even when they meet the requirements. This situation is likely due to the profound stigma against abortion at the social level [5,6], a lack of knowledge by women and health providers of the legal criteria under which an abortion can be obtained, and the absence of sufficient state-level mechanisms to allow women who legally qualify to actually obtain an abortion.

Since the decriminalization of abortion in Mexico City in 2007, accessing safe abortion services in the other parts of the country has become even more difficult for many women. Between 2008 and 2018, 18 states added constitutional clauses that protect the life of the fetus from conception (the 18 states are: Baja California, Chiapas, Colima, Durango, Guanajuato, Jalisco, Morelos, Nayarit, Nuevo Leon, Oaxaca, Puebla, Queretaro, Quintana Roo, San Luis Potosi, Sonora, Tamaulipas, Veracruz and Yucatan. In addition, in 2015, Michoacan presented an initiative, and Chihuahua constitution since 1994 protects life since conception) [7], presumably to prevent similar legal reforms. These states did not, however, amend their penal codes. This means that in these states, the penal codes allow abortion in certain circumstances while the constitution simultaneously disallows abortion in those cases by defining life at conception. This situation has created substantial legal confusion and uncertainty.

Another change that is occurring in Mexico is the increased use of misoprostol as a method of abortion. Misoprostol was originally developed to prevent gastric ulcers but its off-label use as an abortifacient drug has become known worldwide as an effective way to end a pregnancy [8]. However, the pill’s efficacy at inducing abortion depends on it being used correctly—that is, it needs to be taken at the appropriate time during pregnancy and with the correct dosage. Unfortunately, such correct practices cannot be assured in most part of Mexico, where misoprostol is usually taken clandestinely and sometimes without proper instruction for its use [9–11]. In Mexico there is evidence of misoprostol being used as an abortive method as early as the year 2000, and use has increased steadily since then [1,11–13]. By 2009, 29% of abortions in Mexico were believed to involve misoprostol [14]. Women obtain these pills from a variety of sources, but primarily from pharmacy workers, doctors and family members or friends. As a result, the riskier abortion methods used in the past may have largely given way to strategies that while still clandestine, make abortion less dangerous than before. However, no prior research has examined how women’s abortion seeking behaviors (including methods used) vary across the different legal contexts in Mexico, or the impact that misoprostol has had on the approaches women use to access abortion. The purpose of this study is to explore women’s experiences of induced abortion in three federal entities with three very different legal contexts, and investigate if abortion seeking patterns and experiences differ across these settings. To do so, we use qualitative data from women who recently had an induced abortion.

## II. Data and methodology

### Study setting

This study was carried out in 3 states of Mexico: Queretaro, Tabasco and the State of Mexico. These states represent 3 different degrees of restrictiveness of abortion legislation. Queretaro has the “most restrictive” abortion law (exception criteria: rape and guilty by imprudence -imprudencial o culposo, refers to involuntary abortion by the woman or doctor-, Tabasco has a “moderately restrictive” abortion law (exception criteria: rape, guilty by imprudence, to save the life of a pregnant woman, and artificial insemination without the consent of the woman), and the State of Mexico has the “least restrictive” abortion law (exception criteria: rape, guilty by imprudence, to save the life of the woman, and serious genetic or congenital malformation). One noticeable difference between the states is their geographical distance to Mexico City, where women are allowed to obtain a safe legal abortion in the first trimester without restriction to reason. The state of Mexico borders Mexico City, with the capital of the state of Mexico very near to Mexico City (about 1 hour 20 minutes by public transport and 1 hour by car); the capital of Queretaro is located in the center of the country, north of Mexico City (3 hours by public transport and 2 hours by car), and the capital of Tabasco, on the east coast, is very far from Mexico City (13 hours by public transport and 10 hours 20 minutes by car).

Despite the abortion legislation and geographical differences, demographic indicators do not vary much among these states (See S1 Appendix). Tabasco is a more conservative society in terms of their reproductive health behavior. Abortion rates are relatively high in the three states, with Tabasco having the highest incidence of abortion and abortion complications in the country. The proportion of population that is poor in the three states is not negligible ranging from 26 to 37%.

### Data source and data collection

This qualitative study used semi-structured in-depth interviews to collect information from a sample of women with abortion experience. The interviewers focused on women’s experiences seeking an abortion in an illegal setting. A snowball purposive sampling approach was employed. Recruitment of the seed participants was carried out at clinics that provide clandestine safe abortion services; two clinics that meet this criterion were selected in the capital of each of the study states. These clinics provide general sexual and reproductive health services and were accessible to a great majority of the population. In order to be eligible for the study, women needed to be between the ages of 18 and 44 years and belong to the low or middle low socio-economic stratum. Before conducting the interviews with the seed participants and the other participants identified through the snow ball approach, a six-question questionnaire was applied to ensure that the participants belonged to the socio-economic stratum desired. The questionnaire and scores for the socio-economic strata were designed by Asociación Mexicana de Agencias de Inteligencia de Mercado y Opinión (AMAI), and are widely used in Mexico [15]. To diversify the pool of participants, two seed stems were chosen from each clinic, the seeds attended the clinic to terminate a pregnancy and were asked to be part of the study, and if they accepted, an in-depth interview (IDI) was carried out before they were discharged from the clinics or at a later date and at a place of the woman’s choice. At the end of this interview, the woman was asked if she knew other women who had an induced abortion—if she answered yes, she was asked if she would be willing to refer them to the study team. With each new participant, a similar referral approach was followed.

All participants consented to being audio-recorded and the interview time ranged between 45 to 90 minutes. The interviews were conducted in Spanish, digitally recorded, transcribed verbatim, and translated into English. The field team, consisting of two female interviewers and a supervisor, had substantial experiences in conducting qualitative interviews. The team was trained by the authors and the interviewers reported to the supervisor and the authors. Both interviewers carried out the interviews in each of the three settings. They adopted a friendly and non-judgmental attitude to establish ‘rapport’ and ‘trust’ and listen attentively and respectfully to the stories shared by the interviewee. Data were collected between mid-2014 and mid-2015, and the final sample size was 60 (the interviewees’ characteristics are shown in S2 Appendix). Refusal to participate was low (8%). The project was approved by the Guttmacher Institute's Institutional Review Board (IRB) and the MSI Ethics Review Committee (identifier IRB00002197 and 016-12-FT-A respectively).

For the analysis, the Spanish transcripts were analyzed independently using a holistic content approach in order to understand the full portrayal of each interviewee’s life [16]

We first familiarized ourselves with the data by reading transcripts for each state at a time (and hearing the tapes when needed). Then we proceed to develop codes for each transcript of each of the states and identify relevant themes using an Inductive Thematic Analysis [17] through an interaction with the data.

### Limitations of the study

Our qualitative study is not representative of women having clandestine abortion in the three states studied as it is not based on a random sample. However, efforts were made to have a wide representation of middle and low income women in each of the states by starting our snow ball sampling in each state with a seed consisting of 4 initial women (2 women in 2 clinics), who then referred us to other women who did not attended the clinic for the pregnancy termination. In addition, the study is based on self-report of women with respect to their abortion experience and whether or not misoprostol was used. Women may not know exactly what medication and/or dosage was given or used by the provider to terminate the pregnancy.

## III. Results

This section presents findings from four aspects related to the process of abortion seeking behavior: women’s knowledge of the abortion law in their state, the reasons for having an abortion, the methods and providers used and the positive and negative experiences with the methods and providers used. The analysis focuses on the women’s experiences and the similarities across the three settings.

### a) Knowledge of abortion law

Women’s knowledge of the abortion legislation in their state (as well as their knowledge of its legal status in Mexico City) may influence the type of procedure they choose. For instance, in the state with the least restrictive law, women might be better informed of the legislation, know abortion is safe in Mexico City, and might attempt to have a safer abortion compared to their counterparts in the state with the most restrictive legislation where women may have less knowledge of the legislation and safer methods.

In each of the three study states, women have been jailed for abortion. However, the precise number of women jailed in Mexico for having an abortion is unavailable. A BBC report [18] mentions that since 2009 criminalization of women who have abortions increased due to the legalization of abortion in Mexico City (2007): 700 women were reported to have been imprisoned, and another 623 cases are going through the judicial processes. GIRE [19] mentions that around 500 judicial inquiries related to abortion are filed every year, and from 2007 to 2016 there have been 98 women sentenced due to the crime of abortion, but this count is incomplete as only some states report the information by sex.

In our sample, most women thought that abortion was prohibited in their states, (53 out of 60), and in general, respondents’ knowledge of abortion law did not vary by setting. Only a few participants mentioned some specific legal criteria under which terminations were possible, but their knowledge was erroneous: none of the women knew the specific legal criteria for abortion in their state, or the penalties for a woman who had an induced abortion.

What comes out strongly from the narratives of the women in all three settings is their fear of being jailed for having an induced abortion.

Sofia, from the State of Mexico, said even to tell her story made her scared:

***Participant*:** *I know [abortion] is prohibited*. *In fact*, *I was a little frightened*, *because I thought that if I told my story I will be taken to jail*. …

*(State of Mexico*, *P2*, *30 years*, *complete university*, *consensual union*, *no children*)

Similarly, Erika narrates her impression of the legal situation of abortion in Tabasco:

***Interviewer***: *You told me that here in Tabasco*, *abortion is clandestine*. *Why*?

***Participant*:** *Because the procedure is not offered in any clinic*. *Here*, *a person could give you a concoction to drink*. *But if somone accuses the woman*, *well she could go to jail—I am refering to the woman that gave the concoction*.

***Interviewer*:** *And what happens to the woman that sought out the abortion*?

***Participant*:** *Also goes to jail*.

*(Tabasco*, *P6*, *23 years*, *primary*, *consensual union*, *1 child*)

Despite the extensive media exposure of the legalization of abortion in Mexico City (the case was taken to the high court of the country, and sessions in favor and against the legalization of abortion in Mexico City were nationally televised), only one third of the women in the study were aware that abortion is legal in Mexico City. Thus, women in our sample from the State of Mexico tended to be more aware of the legality of the procedure in Mexico City, most likely because of the proximity of the city to them.

Laura’s narrative below represents the voices of the women who knew about the change of abortion legislation in Mexico City.

***Interviewer*:** *Do you know if it is legal in D*.*F*. *[Mexico City]*?

***Participant*:** *Yes*, *I heard of it and I think that is very good*….

***Interviewer*:** *Is there any extraordinary case where it is legal [here in the State of Mexico]*?

***Participant*:** *I do not know*. *I believe that for rape*.

*(State of Mexico*, *P5*, *32 years*, *incomplete high school*, *separated*, *3 children*)

### b) Reasons for abortion

The reasons why women choose to have an abortion were varied, with no clear patterns across the three states. The most cited reasons were economic reasons -almost two thirds of the women-, followed by problems with partner -almost half of them-. Other reasons were mentioned, including not wanting to have a child at that moment or none at all (spacing or limiting), not wanting to be a single mother, too old to be a mother, not being in a committed relationship, and wanting to continue studying, among other circumstances. In two cases (one in the State of Mexico and another in Tabasco), the women were raped and that was the reason they sought an induced abortion. In about half of the cases multiple reasons were mentioned.

*Economic reasons*, which were the most commonly mentioned, were present in many of the narratives. For example, Carmelita from Tabasco felt her financial situation was already too strained to add another child.

***Interviewer*:** *You started to suspect you were pregnant*?

***Participant*:** *Yes*, *I bought a pregnancy test in the pharmacy and was positive*. *When I found out I was pregnant again*, *I sat down on the bed and started crying*, *just thinking how I would manage another child*. *There are many problems*. *We do not have enough money and all four of us live in one room*. *There is a room*, *another for the kitchenet and the bathroom*. *The rent and everything else was too much and I felt everything was coming down on my head* … *Terminating the pregnancy was the only solution*.

*(Tabasco*, *P17*, *30 years*, *secondary*, *married*, *2 children*)

Clara from Queretaro was also concerned with economic reasons:

***Interviewer*:** *When you found out*, *you discussed it with your partner and what did you talk about at that moment*?

***Participant:** Mainly, that it was not wanted, … we had other plans, we talked and agreed that it was not wanted, and that our economic situation was not that good as to have a second baby and said not now, both of us were in agreement and that is why we interrupted it*.

*(Queretaro*, *P11*, *21 years*, *complete secondary*, *consensual union*, *1 child*)

Another very common reason in the three states for not having the child and seeking an abortion was the *bad relationship* women had with their partner. Some partners were violent to the women, while others had drinking or drug problems or were unfaithful.

Sofía, for example, said that she sought an abortion because of the bad relationship with her partner:

***Interviewer*:** *How did you feel when you knew that you were pregnant*?

***Participant*:** *It was nice*, *but in that moment many things come to mind*, *starting with the problem I had with him*, *that he was a drug addict at some point*, *he drinks*, *and he was going to harm me*, *and I saw what was going to happen to me*. *And I said no [to the pregnancy]*…

*(State of Mexico*, *P2*, *30 years*, *complete university*, *consensual union*, *no children*)

The narratives of women show, however, that *reasons for abortion are multiple and interrelated*. The case of Julieta is an example of how different considerations enter into decision-making to stop a pregnancy. In her case, her decision was influenced by economic reasons, her relationship to partner, and not wanting her two children from her first marrige to have a half brother or sister.

***Participant*:** *… We were in a dificult economic situation*.

***Interviewer*:** *How were you emotionally*?

***Participant*:** *Emotionally well*, *but economically very difficult*. *Sometimes we did not have any money*. *When I realized I was pregnant I got scared*. *My children … are at school*. *… I have always decided not to get pregnant for another person*, *I did not want them [the children] not to be [full] brothers*. *… maybe they might have enjoyed being together*, *but I was selfish … Maybe things would change [with new partner]*, *and we might not be so close*, *sometimes children separate couples and there are problems* …

*(State of Mexico*, *P7*, *39 years*, *complete high school*, *consensual union*, *2 children*)

### c) Method and providers of abortion

Women’s selection of abortion methods and providers involves many issues, including economic consideration, knowledge of the type of methods available and knowledge about where to obtain them, among other. In some cases, there is really no selection of the method and/or provider because only one is available or accessible to women. This section describes the methods and providers used by the participants and how they chose the method and/or provider.

### Methods

Among participants in Queretaro the main method used was misoprostol (17 out of 20 women). Limited use of other methods was reported, including MVA. All participants in this state succeeded in having the abortion with one attempt.

In the State of Mexico, misoprostol is also commonly used among participants (15 out of 20 women), but it is used less as the sole method than in Queretaro. Fewer women in the State of Mexico used MVA than in Queretaro, and while no participants in Queretaro used D&C, some did in the State of Mexico. In the State of Mexico, 4 women made two attempts to complete the procedure, as the first attempt failed.

Participants in the State of Tabasco presented a very different scenario. Still, misoprostol was the key method used for abortion (10 of 20) and it was commonly used among the participants to self-induce the termination. But in most cases, it was used in combination with another method, usually herbal teas, drinks, remedies, traditional medicine. Possibly, incorrect usage of misoprostol (too low dosage) is prevalent in this state as around half of the women completed the abortion in one attempt, a larger number of women had to have a second attempt to achieve the abortion, and even one woman had a third attempt to finally terminate the pregnancy. Often, the traditional method was provided by a traditional midwife. Also, in many cases, the women seeking abortion did not know whether other medicines, such as misoprostol, were added to the drink and the amount added to the concoction.

The methods chosen by women were usually those recommended by close friends, relatives or partners, those who they trust. Only in a few occasions did women depend on the internet as the source of their methods. The case of Gabriela represents those of many women in the study. She took the decision to terminate the pregnancy; she told her sister who advised her to use misoprostol, guided her on how to use it and stayed with her during the abortion process.

***Interviewer*:** *Did you know how you were going to interrupt it*?

***Participant*:** *With pills*.

***Interviewer*:** *How did you get the idea of the pills or who told you*?

***Participant*:** *With talks with friends*, *I know that these pills were abortive and my sister Rose confirmed it*.

***Interviewer*:** *Did they tell you how to use them and the cost*?

***Participant*:** *Rose told me how to use them*, *and the cost I found out when I went to buy them* …*in the Pharmacy Ortiz…they sell them each pill separately*.

***Interviewer*:** *How did you take them*?

***Participant*:** … *2 vaginal and 2 oral…*. *And another pill the next day* …*in case there was any residual or something left*.

*(State of Mexico*, *P13*, *25 years*, *complete university*, *married*, *1 child*)

In Queretaro and the State of Mexico, some of the participants who self-induced the abortion with misoprostol got help from an NGO. The adequate advice women received from knowledgeable NGO providers led to the women having successful and safe medical abortion terminations. Clara’s experience represents the stories of women who self-induced with misoprostol with the help of an NGO. She did not know what to use or do to terminate the pregnancy, so she talked to her friend who introduced her to one of her friends who took her to Gloris who worked in an NGO focusing on of reproductive health and safe abortion. There she received help on what method to use, what she should expect from the procedure and the safety and steps she would need to take, such as having an ultrasound before and after the pregnancy termination, a 24-hour hotline support, and counsel with respect to whether she really wanted to have the abortion.

***Interviewer*:** *What options did you [the woman and your partner] consider for the [pregnancy] interruption*?

***Participant*:** *Well*, *we needed to research with what and with whom and to be safe* … *that was not high risk*, *that nothing happen to me…I have a friend and she took me to Gloris who help me [with the abortion procedure]* …

***Interviewer*:** *Who is that person*, *a doctor*, *…who is Gloris*?

***Participant*:** *No*. *A friend of my friend*, *took me to Gloris [a worker] with an NGO*. *She told me it was safe and they could advise me and I could receive information about that*, *so I went*.

***Interviewer*:** *So that was your first step*, *to go to the NGO*. *Did you search for another alternative*?

***Participant*:** *No*, *just with her directly*, *because they told me that it was safe and reliable*.

*(Queretaro*, *P11*, *21 years*, *complete secondary*, *consensual union*, *1 child*)

Participants in Tabasco who self-induced the abortion using misoprostol combined with traditional methods—concoction and other traditional methods–generally had experiences that were similar to that of Maria Fernanda. She and her partner choose to use misoprostol through the advice of friends. However, in this case, the information given was to take misoprostol with coconut water in the morning before breakfast. The method did not work, so they resorted to a second attempt, using traditional methods obtained through a traditional midwife.

***Interviewer*:** *So both of you [participant and husband] agreed to interrupt the pregnancy*, *and did you know how to do it*?

***Participant*:** *My husband had friends who had situations similar to ours*. *My husband’s friend and his girlfriend did it [aborted]*. *He bought the Cytotec*, *I think that is the name*, *and told us he was going to get it*. *… His friend got them… I think his friend had a relative nurse and it was much easier to get it [the cytotec]*. *Because*, *if I went to a pharmacy they would have refused to give it to me*.

***Interviewer*:** *Why*?

*Participant*: *Because abortion is not legalized*. …

***Interviewer*:** *Did the friend tell you how to use it*?

***Participant*:** *Yes… There were 2 pills…Before breakfast I took 2 pills with coconut water*…

***Interviewer*:** *Why with coconut water*?

***Participant*:** *I do not know*, *but I just did what they told me*, *and I drank it*.

***Interviewer*:** *What did you do later*?

***Participant*:** *… I started with a little pain*. *…*. *but did not have an effect and we search for other methods*. *… As I know some traditional midwives*, *and I had visited one of them…*.*I went with her [to the house of the traditional midwife] for the abortion*.

*(Tabasco*, *P12*, *20 years*, *high school*, *1 child*)

### Providers

Some women choose to self-induce the abortion not consulting or going to a provider. However, other do choose to go to a provider to have the abortion termination. The providers most commonly resorted to are gynecologists and traditional midwives. Among participants in Queretaro and the State of Mexico, the gynecologists they sought were very diverse and different in each of the study areas, for example, some terminations were done by gynecologists well trained in abortion in a legal setting, others convinced their gynecologists to perform the abortion and others found a gynecologist that advertised their services in an illegal setting In rare occasions was a nurse or pharmacist sought.

In the state of Queretaro and the State of Mexico some participants (5 and 4 out of 20 respectively) had a safe abortion in Mexico City. This is not surprising since both states are not far from Mexico City. The providers of these abortions were gynecologists who were well trained in abortion methods and worked in health facilities (public and private clinics) that were known for providing high quality abortion services. The providers sought by participants in the state of Tabasco presented a completely different pattern than women from the other 2 states. No woman went to Mexico City to have a safe legal abortion, which may be understandable as the state is farther to the capital of the country relative to the other two states; it takes about 13 hours by public transportation to travel from the capital of Tabasco to Mexico City. None of the 20 participants in Tabasco sought an abortion from medical personnel (doctor or nurse). Instead, women either self-induced their abortions or seek the help of a traditional midwife to terminate the pregnancy.

In Tabasco, also the provider they resorted to came from the advice of close friends/relatives/partner. But a few participants, like Cynthia, followed their neighbors’ foot-steps by using providers that the neighbors had used. In this case, Cynthia’s neighbor took her daughter to a traditional midwife for pregnancy termination. For her own abortion Cynthia decided to also go to the same traditional midwife.

***Participant*:** *So he [husband] asked me what are we going to do*. *And I told him that I knew a traditional midwife that gave home remedies*.

***Interviewer*:** *How did you know her*?

***Participant*:** *She is known*. *She is not a friend…a neighbor … took her daughter had aborted*, *through her [traditional midwife]*…..

***Interviewer*:** *Where do the remedy and procedure was done*?

***Participant*:** *In her house [traditional midwife]*. *I went with my husband*. …

*(Tabasco*, *P20*, *34 years*, *complete high school*, *married*, *3 children*)

Participants in Queretaro and the State of Mexico, who went to Mexico City to have a legal safe abortion relied on the internet. Some did not know that abortion was legal in Mexico City, but when they searched on the internet and found that abortion was performed legally they felt that was the best option for them. Others heard from a friend or someone else close that abortion was legal in Mexico, then they used the internet to investigate more. They became convinced that it was better to have the abortion in a place where it is legal, as they believed it was going to be a safe abortion. When Emma needed to have an abortion, she and her friend discovered on the internet that abortion was legal in Mexico City. They both preferred the option of having the procedure where it is legal.

***Participant*:** *…*.*She [friend] was pregnant when she was young and I was with her when this happened and I knew what she did [to stop the pregnancy]*, *but I never knew the name of the injections and where she got them*.

***Interviewer*:** *You did not have the contact to get them*?

***Participant*:** *No*, *because they were given by a friend of her ex-boyfriend and he gave them to her… My friend told me to search on the internet for the injections*, *so we [friend and participant] did*, *… and we found various places where abortion could be done*, *free and legal in Mexico City*. *I did not know this … There were several hospitals from the government* …*So we went [to Mexico City]*….

***Interviewer*:** *In your search on the Internet*, *did you find any other method*? *Why did you go to DF [Mexico City]*?

***Participant*:** *Because*, *really*, *I did not know what to do*, *and because I saw it was legal*, *I was frighten to go to another place… But first*, *I decided to go to the Health Center [locally in her state]*, *… to confirm that [in Mexico City] it was allowed [legal] and to learn if the pills [misoprostol] worked*.

*(State of Mexico*, *P11*, *23 years*, *incomplete high school*, *consensual union*, *no children*)

In summary, methods and providers chosen usually come from the advice of a close friend/relative/partner, those who they trust. Misoprostol is used in the three states extensively, either alone or in combination with other methods. But there is a gradient of use by the state of the participants: it was mostly used by participants in Queretaro, followed by those in the State of Mexico, and then by those in Tabasco. In contrast with the other settings where no woman went to a traditional provider (i.e. the State of Mexico) and where only 2 women did (i.e. in Queretaro), all participants in Tabasco that went to a provider to have an abortion used traditional midwives. No medical personnel was sought for abortion by participants in Tabasco. The method and provider used for abortion are relevant for successful termination of pregnancy. It is therefore not surprising that in Queretaro, all participants succeeded in having their abortions with one attempt; in the State of Mexico, four women made two attempts to complete the procedure–i.e., two methods were used sequentially before the abortion was completed; and in Tabasco, 8 participants had to have two attempts to complete the procedure, and one had to have a third attempt.

The findings suggest that the degree of restrictiveness of the abortion legislation does not relate to the type of method and provider used, and to the safety of the procedure. On the other hand, proximity to Mexico City, where abortion is legal, has the beneficial effect of obtaining a legal and safe abortion for women.

### d) Women’s positive and negative experiences with method and provider used

Women’s abortion experiences varied widely depending on the method they used and the provider used (if any). In the following section, we highlight four types of providers and methods to illustrate the diversity of women’s experiences in an illegal setting: 1) self induced abortion by the women using misoprostol; 2) women seeking abortion with traditional midwives using misoprostol and other remedies; 3) women obtaining abortions from doctors in an illegal setting and 4) women having legal abortion in Mexico City. Women using the same type of method and provider may end up having different outcomes, one with a positive experience or a safe abortion and the other with a negative consequence or an unsafe abortion.

#### d.1. Experiences of women who self-induced abortions using misoprostol alone

Although the use of misoprostol was common among the study participants, the stories of women using this method were often dissimilar. In part, this is due to variation in the doses of the misoprostol used, which were often incorrect. The women did make efforts to find the correct approach and doses of misoprostol use for an abortion. They all searched for information with their friends, on the internet, and with relatives, among others. But the information given was not always adequate. The women ended up following the information given by who they trusted most, or that sounded the most reliable, and this process often led to use of incorrect doses.

Daniela, from Queretaro, self induced the abortion with misoprostol and had a sucessful story. She obtained the information from the Internet and her partner was with her all the time:

***Participant*:** *… I used first the pills as instructed … The pain was very strong … I took camomile infusion to calm the crapms that were stronger than a regular menstruation …*. *I lied down in bed*. *Then come the first colic*, *then another*, *… I started at 5 in the afternoon of Saturday and by one in the morning it was finished and I slept well* … *There was no pain … I had a pap smear test and everthing was fine*. *Then I went to medical revision and was fine … I did not have complications*, *only bleeding*, *but I did not worry because I was informed that was ok* …

*(Querétaro*, *P1*, *25 years*, *incomplete university education*, *single*, *no children*)

A very different story was narrated by Paola. She ended up in the hospital to complete the self-induced abortion with misoprostol and had a D&C with local anesthesia. A friend told her to take four pills of cytotec to abort (two vaginally and two swallowed).

***Participant*:** *… She gave them to me* … *because her daughter had an abortion and these were pills left over*. *She said not to be scared*, *that I would feel like vomiting*. . . . *She guaranteed me that nothing was going to go wrong and I believed her*, *that there was going to be some bleeding*, *and I would feel like nausea … That I was going to feel like if I was sick in the stomach*. *… Back in my home*, *I took the pills*, *went to sleep*, *and at about 4*:*15 am a strong contraction woke me up*. *… I threw up all that I ate… Later on*, *I woke up and I was all wet with blood … I used a sanitary towel*, *but when I woke up again I was in a puddle of blood…*.*I called my friend to come… We went to see a doctor friend of hers … and he called another friend Dr*. *John who was at a Hospital in Toluca*. *… The doctor arranged for me to be hospitalized*. *I was told to say that it was a strong mentrual bleeding* … *Because of the amount of blood*, *he told them it was urgent to take me to the operating theater and have a D&C…*.*The pain was still severe*. *They used blockage* …

*(Estado de Mexico*, *P8*, *42 years*, *technical career*, *separated*, *2 children*)

One factor that often led to a more positive experience in our participants’ cases was being guided through the process by an NGO. These stories were common; women found the NGOs through referrals from friends or the internet, and the NGOs’ staff advised them to have an ultrasound, gave them the pills, instructed them on how to use them, informed them what they would experience, and told them to have a follow up ultrasound and a doctor visit. What was more important to many women, however, is that staff of the NGOs were with them during the procedure by phone, reassuring them; in addition, the doctors the women visited for the ultrasound and the followup visit were often friendly doctors that wanted to help women in this illegal setting.

Cruz told us her story.

***Participant*:** *… I had an ultrasound that the NGO recommended to check everything was fine… I was given information regard [pregnancy] interruption and some pamphets of the association*. *I was informed how to take the treatment [cytotec]*, *which was the best form … they talked about the cramps … I had cramps*, *I had diarrhea*. *The cramps were stronger that usual cramps*. *And I took the painkiller they told to have if in need… I did not feel any problem and everthing happened as they said it would*, *how much I was going to bleed*, *large blood clots*, *and I would bleed more days that a menstruation … There were no complications*. *Everything was normal*. *They recommended a check up which I did … The doctor said everything was fine*.

*(Queretaro*, *P4*, *27 years*, *complete university*, *consensual union*, *0 children*)

#### d.2. Experiences with traditional midwives using misoprostol and other remedies

Seeking help from traditional midwives was more commonly reported in Tabasco than in the other studied areas. Narratives of abortion procedure done by traditional midwives were found to be both positive and negative, and ranged along the spectrum of safety. Many of the traditional midwives used misoprostol.

The case of Carmelita who lived in Tabasco, is similar to those of other women who relied on traditional midwives to terminate the pregnancy. Most women reported some familiarity with the traditional midwives, as they are birth attendants and the provider of traditional medicine in the community. Usually, women seeking an abortion stayed at the traditional midwife’s home during the procedure. The traditional midwife cares for the woman throughout the process, and some of them give antibiotics and medicine for the pain. However, we do not know with precision the dosis used of misoprotol inserted in the vagina and if the concoctions also had misoprotol, but in this case the experience for the woman perspective was positive.

***Participant*:** *…* .*I went to my aunt–a traditional midwife- and explained to her the situation*, *and she told me to buy pills [cytotec] … however*, *to buy them a doctor’s permision was needed*. *But she said I should not worry as she had a friend that could get them*. *That I should only give her the money [traditional midwives do not tell how many pills they are going to use*, *but they request payment] … She [traditional midwife] administered this concoction with several herbs in her house [traditional midwife’s home]*. *… At 8pm*, *she told me to drink it [the tea]*. *This [taking the pills] has to be done in the night because you lie down and the body works*. *She [the traditional midwife] introduced the pills in the vagina and another I drank it with a concoction … It tasted horrible*. *I drank it… I lied down and every half hour I had another strong concoction with cinammon*. *This [the concoction] was going to help to get rid of everything … At midnight the pain started*. *Like if I was having a baby*, *and I was only 1 month and 15 days pregnant*. *… There was a lot of bleeding*, *…*. *I did not sleep because I was scared… of seeing so much blood …*. *My aunt kept on changing me*. *At 5 in the morning I threw a ball*, *…*, *it was a placenta*. *… I felt it very clearly how it came out … There were clots of blood … Like a ball of blood*. *My aunt said that was the baby*. *The bleeding calmed down at around 6 or 7am*, *and gave me an injection to clean me from inside … Then she gave me camomile tea with a boiled cactus and other leaves*. *That tea is for cleaning everything inside and prevent an infection*. *I stayed there until the afternoon*. *I recovered and I felt normal*. *I was not bleeding much*, *it was like a menstruation*. *I left and went with my mother and stayed with her 3 days to recover*.

*(Tabasco*, *P17*, *29 years*, *complete secondary*, *married*, *2 children*)

However, not all women who went to traditional midwives had such a good experience. A few women who went to a traditional midwife reported that they were massaged. In one case the midwife introduced her hand in the uterus to remove the placenta and any remains of the fetus. The narrative of Mary from Tabasco demonstrates how bad an experience and unsafe an abortion through a traditional midwife could be. The result could have been fatal, but in this case it was not, but certainly it was very unsafe abortion.

***Interviewer*:** *… my sister-in-law gives birth with the help of a traditional midwife*, *she told me that this person could help me*. *… I arrived [at 10am] and explained to her the situation and she said she would prepare something … I did not know what the drink had … It tasted bad … She told me that after a few hours I was going to feel bad … that I should go home and around 12 or 12*:*30 to return to her when I had pain which I did … I started with cramp pain …*. *It became strong … And I started to feel bad and went to the toilet and it came out … There were clots*. *… The midwife introduced her hand [in the vagina] and she pulled out the placenta*.

***Interviewer*:** *Did it hurt*?

***Participant*:** *Yes*. *I was feeling very bad and she gave me alcohol [to smell] to revive me*.

***Interviewer*:** *And … how long did it last [that she had her hand inside you]*?

***Participant*:** *About half or 1 hour*. . . . *I was very dizzy*. *My mother-in-law was behind me and was giving me the alcohol [to smell]*. *And it started hurting more* …

***Interviewer*:** *And then what happened*?

***Participant*:** *She put the hand inside and took our something and put it in a bag*. *And started washing me with warm water and I stayed for a while lying down on the bed*.

***Interviewer*:** *Did you feel better*?

***Participant*:** *That day I felt very weak … And I stayed there to recover*. *At around 3 or 4 in the afternoon I went home*. *… I was feeling very weak* … *I took a taxi home*. *… [At home] I stayed laying on bed … and next day I walked and I was bleeding a lot*, *but it stopped*. *… It was like a menstruation for 4 days*.

*(Tabasco*, *P14*, *23 years*, *incomplete primary*, *consensual union*, *3 children*)

#### d.3. Experiences of abortion performed by doctors in an illegal setting

While many would assume that medical doctors perform safer abortions than other providers, this is not always the case in our sample. Several women from Queretaro and State of Mexico chose to have an abortion in their area with a gynecologist believing the procedure would be well performed—here we present two contrasting cases of women who went to gynecologists who advertised doing abortions in an illegal setting. In both cases the same method was used, misoprostol and MVA with paracervical local anesthesia, but the women’s experiences were radically different. A paracervical block is an anaesthetic procedure used in obstetrics and gynecology, whereby a local anaesthetic is injected into between two and six sites at a depth of 3–7 mm alongside the vaginal portion of the cervix in the vaginal fornices. For more information on this topic refer to findings from a randomized controlled trial of paracervical block for pain control in first-trimester surgical abortion [20].

Karina described the procedure, the extreme pain she experienced and her inability to walk after the procedure, characteristics associated with perforation of the uterus.

***Participant*:** *He was a …*. *gynecologist…*. *He explained to me that he was going to put a vaginal catheter*, *and an intravaenous solution… and he was going to give me some pills[misoprostol]… to dilate … he was going to give me anestesia* … *and he was going scratch and then remove it [the product] [MVA]*. *… I was not going to have any pain and there was going to be no problem and after*, *there was going to be some bleeding for a week and that was all*. *That everything was going to be normal … They put in me intravenous solution*, *… they gave me pills … I did not feel anything [at that time]*. *… they put me like in a hospital stretcher that had some things to leave the legs hanging*. *He told me it was not going to hurt*, *that it was a prick and that they will put an anaestesia which he did*. *… But it was horrible*. *The pain was undescribable*. …

*I was given two prick [in the vagina] … I shouted a lot … I remember that he said that in five minutes the anesthesia will make effect and he left [the room]*. *… I was in great pain … In the abdomen … After five minutes he came back to the room* …

*The pain was unbearable … and he said he believed that I was ready … I felt he was scratching*, *… it hurt everything … and I cried and I cried and shouted because the pain was horrible*. *It is the strongest pain I ever experienced … Then he … was like sucking somethig*. *It was an awful situation and I was in a lot of pain*. *… It lasted less than 10 minutes… I kept on telling him to stop*. *I wanted to go*. *…*. *He was a bad doctor*, … *then he finished*, *but the pain continued very strong*. …

*It hurt me the whole body, the legs, the abdomen, the arms. I kept on crying a lot, and could not stop… I only wanted to leave and get home, lie down and did not want to think of this [experience]. … It is the worse experience I have had in my life … The pain was unbearable. … I tried to stand and I could not walk. … He gave me a prescription for antibiotic and medicine for the pain*, …

***Interviewer*:** *For how long he gave you the antibiotic*?

***Participant*:** *… I think every 8 hours for five days*, *and the other medicine for the pain every 6 hours for three days*. . . . *I could not walk*, *nor take the bus*, *so we [her mother and her] had to take a taxi … I was too tired* …

*(Queretaro*, *P17*, *23 years*, *high school student*, *single*, *0 children*)

In contrast, April’s experience with a doctor who advertised his services in an illegal setting was positive, with no pain and no complications.

***Participant:** [I was] Six week … the gynecologist … gave us an appointment … He started to ask why we wanted to abort. And I told him that 3 month ago I had a baby. He told me that yes, there was the posibility that he would do the abortion, but the cost was $7,000. And that we should decide if we accepted or not before he explain to us [the procedure]. My partner said yes. … The gynecologist explained to us that there were two forms to abort. One with pills, you go home and do it alone… The other is to abort with him and he will use suction. … I told him it was more convinient that he would do it …. he told me he will do it next day … And gave me two pills [of misoprostol] … one to take in the morning by mouth and one vaginal and stay resting. … He said I should not be left alone, that I was going to feel warm, and maybe sweat and if the bleeding started I should come to him immediately … my partner stayed with me, did not go to work … I started feeling warm in excess and I started bleeding slightly …. and my husband took me to the gynecologist*.

*… I was scared*, *… He put me in a stretcher*, *…*, *I was given a hospital gown*. *He told me it was not going to hurt much*. *He put me on anesthesia*.

***Interviewer*:** *With intravenous solution*?

***Participant*:** *… No*, *he did not inject me*, *but he put a liquid through a vaginal foley … and there he injected a sedative or a liquid*. *I was crying because I was worried for my health*, *as I have never done anything like this … But the doctor told me to be calm*, *that I was going to be fine*. *That I was going to feel very little pain and then I was not going to feel anything*.

*Then he inserted a tube … and I saw a hugh syringe that he inserted*, *and then I did not feel anything from the waist down*. *… I did not see but my partner saw everything*…

*He saw how he put the pliers, they open the lips and then was scratching the uterus so the the tube would enter to suck out* …

***Interviewer*:** *How long did the procedure lasted*?

***Participant*:** *Abou 6 or 7 minutes*. *… it was very fast … Then he took out the splits*, *he clean me*, *showed the blood and the pieces he took out to my husband … I did not want to see … I laid down and later I left walking*.

***Interviewer*:** *Did he give you any medicine*?

***Participant*:** *Yes*, *he gave me some pills for the pain and headache*, *… and ask me to take a week rest and to return in about a week for a checkup*…

*(State of Mexico*, *P10*, *23 years*, *complete secondary*, *consensual union*, *1 child*)

#### d.4. Experiences with legal abortion in Mexico City

Some women from Queretaro and the State of Mexico decided to have a legal safe abortion and went to Mexico City, in abortion clinics with an approved medical provider. Most women went to private clinics, but a few attended Mexico City government facilities for services free of charge. For the women who opted to go to Mexico City to have a legal abortion, the experience was positive. In both, private and public clinics, they received clear information from the doctor about the procedure and what they would feel. They were treated in a positive way and they were even given a hot drink at the recovery room and asked to wait until fully recovered and ready. They were given preventive medicine like antibiotics and pain killers, and did not have complications; in addition, the clinic had a 24 hours service line where they could call if they had a problem. Here we present two cases, one of the women that choose to go to a private clinic in Mexico City and another that was helped by an NGO to attend a government facility that provides free services for women in Mexico City.

Maria Jose is from Queretaro, and was originally planning to have the abortion in her state. However, after a bad experience with fake providers of abortion in Queretaro (pro-life centers advertising that they do abortions) she decided to seek the abortion in Mexico City where it is legal. She narrates her positve story:

***Participant*:** *… I looked for [abortion] clinics in Queretaro*. *… I went to one … [they were prolife and] she tried to convince me not to make it*. *That my live was in danger*, *so as the live of the baby*, *… I left … And I thought everything was going to be the same in Queretaro*. *So I decided to seek [an abortion] in D*.*F*. *… we look in the internet … and I choose a cheaper one*. *I told them I was from Queretaro and needed an interruption*. *They asked me if I wanted an appointment*. *I told them that I had to go and return on the same day and that I wanted them to explain me how it would be [the procedure]*. *They told me it was aspiration with local or general anesthesia … I told them I wanted general anesthesia*, *and wanted to know how long the procedure would last*. *… If it was the general anesthesia*, *it was going to be 2 or 3 hours*. *… to feel well and that I could take a tea*, *and wait until I feel better*, *and to arrive fasting*, *and that I was not going to feel anything and the next day I would just have light pain and little discomfort … They gave me the appointment for 10am* … *I had general anesthesia and … After the anesthesia went off*, *I felt a little discomfort*. *I had like a heavy menstruation and I waited for the anesthesia to finish*. *My friend was with me all the time … When the effect of the anesthesia was gone*, *they took me to a lounge and gave me tea*. *… I recovered … I stayed like 1 ½ hour*. *Then my friend took me to the bus station to go back to Queretaro*.

***Interviewer*:** *Did you have a subsequent complication*?

***Participant*:** *No*. …

***Interviewer*:** *Did they give you some medication or prescription*?

***Participant*:** *Yes*, *they gave me a prescription*. *They gave me antibiotic and medicine for the pain*. …

***Interviewer*:** *Do you think the service was good*?

***Participant*:** *The truth yes*. *It was very clean*. *The clinic was very pretty*. *It was a new house*, *it did not look like a hospital*. . . . *The doctor explained to me everything from the beginning how it would be*, *what I was going to feel*. *Everything went very well*. *I was lucky to find a good place*, *and that everything went well*.

***Interviewer*:** *Did they give you an open line in case you had a complication*?

***Participant*:** *Yes*, *they gave me the phone number for the clinic*, *a switchboard and from there they could reach the doctor 24 hours a day*. *I did not have to phone*. *I felt very well after the procedure [no complications]*.

(Queretaro, P9, 33 years, complete high school, single, 1 child)

This section clearly exemplifies the difficulties women encounter in quest of a safe abortion and a good experience having the procedure, from the perspectives of our study participants. Their stories are diverse and the experience of pregnancy termination may be good or bad even using the same method and type of provider. Although, some of the women may not know for sure the qualifications of the providers or the methods used, in general, the narratives provided seem plausible in the context of restrictive abortion laws. Legal restrictions on abortion put women in a difficult situation. While they generally do not prevent abortions from taking place, they make it difficult to ascertain the quality of abortion services or the reliability of the provider, as the health providers could act as impostors. Restrictive abortion laws, result in providers not being accountable to norms or WHO standards [21–24]. Furthermore, seeking a knowledgeable health provider with the right skills to perform an abortion is difficult because of the clandestine environment and the lack of liability for their work. Furthermore, self-inducing the termination of pregnancy by women with the correct procedure and doses is not guaranteed as information is often not available. Women tend to trust the information provided by their close friends and family, who do not necessarily have correct information regarding methods and providers. A relevant finding is that all the women who went to Mexico City to have a safe abortion had a good experience. Similarly, women who self-induced the abortion with the help of an NGO who gave them correct information about misoprostol use and supported them during the process had good experiences.

## IV. Discussion and recommendation

Mexico, characterized by abortion legislation specific to each state, and where the law is restrictive in all of them with the exception of Mexico City, provides the opportunity to compare abortion seeking behavior among states with different levels of restrictiveness of abortion legislation. In this exploratory study, the assumption considered is that where legislation is more restrictive the options of having an abortion with safer methods and providers would be more limited and those available would be riskier compared to those in states with less restrictive abortion legislation. Findings from this study about the condition under which women have abortion contribute to the scarce literature on the topic in Mexico and Latin America.

Regarding women’s knowledge about abortion legislation, no difference is found among participants by state or level of legal restriction. Most women thought that abortion was prohibited in their states, but did not know the legal criteria under which abortion is allowed. Only one third of our respondents knew that pregnancy termination was allowed in Mexico City. A general feeling among them was the fear of being jailed because of having an abortion, and despite this apprehension they sought the procedure. As mentioned by other investigators and shown by our qualitative data analyzed [25,26] restrictive laws do not eliminate the practice of abortion, women continue to seek abortion in legally abortion restrictive settings.

Women seek an abortion for various reasons. Research on the topic has cited economic reasons as a common motivation for seeking an abortion in many LDCs, but women also report multiple and overlapping reasons for ending an unintended pregnancy [25,27–30]. Findings from our study supports the available literature and women in the three states present similar reasons. The economic reason is the most commonly reported for having an abortion. Many other reasons were mentioned, and nearly half of them gave multiple reasons for seeking an abortion in each of the states examined.

Various abortion methods and providers are reportedly to be used in each of the three study areas. However, similar to the findings in other studies (e.g. [25]), evidence from the three study states suggests a high use of misoprostol which appear to be on the rise. Studies have indicated that in highly restrictive contexts, clandestine abortions are now safer because fewer occur with the use of dangerous and invasive methods [25,30], and our findings support this evidence as women do not reported the use of more invasive methods (insertion of hangers, etc.).

Misoprostol seems to be used extensively for abortion is the three states, either alone or combined with other methods. But there is a gradient of use by state: the medication is used more in Queretaro, followed by the State of Mexico, and then by Tabasco.

Our findings indicate that there is a new pattern of abortion service provision in Mexico where misoprostol plays an important role, changing from no use of misoprostol before 1995 [9,31] to 29% using the method among women seeking abortions in 2007 [14], and to the vast majority of women currently using misoprostol, as shown in this study. [14] However, we found that the use of misoprostol may not have concomitantly increased the safety of the procedure. For instance, women usually do not have the accurate information on how to safely self-induce an abortion: informants about misoprostol might not also have the correct information (on the dosage, when and how to take the misoprostol), and to obtain the correct information seems to be fortituous. Furthermore, some providers who use the method often recommend different dosages and approaches of introducing the pill into the body and some use it along with concoctions, including traditional herbs, teas and other substances.

Our findings indicate that there is no clear relationship between the restrictiveness of abortion legislation and the safety of abortion (including use of appropriate methods and providers). In Queretaro, the state with the “most restrictive” abortion law, all participants completed their abortions in the first attempt, 12 women self induced the abortion and some of them had good information about the use of misoprostol, and 5 went to Mexico City to have a legal abortion with a trained provider. In the State of Mexico, with the “least restrictive” abortion law, 4 out of 20 women did not achieve the abortion in the first attempt. Five women self induced the abortion, and a large proportion of them went to a gynecologist, 4 of them went to Mexico City to have a legal abortion with a trained provider. The State of Tabasco with a “moderate restrictive” abortion law, has the poorest pathway to abortion, no medical profesional was involved in the abortion procedure, mainly women self induced or the abortion was performed by traditional midwives. Eight women completed their abortion in 2 attempts and 1 woman in three attempts, no woman in this state went to Mexico City to have a legal abortion. Two factors seem to explain the lack of association between restrictiveness of abortion law and the safety of abortion in our study. It seems evident that being near Mexico City, where abortion is legal up to 12 weeks, has had a beneficial effect for women living in the 2 states that are nearer to Mexico City (Queretaro and the State of Mexico). Secondly, information about how to obtain and use safer abortion methods and providers seem to diffuse better among women in Queretaro and the State of Mexico than in Tabasco. These two reasons tend to produce, at least partly, better abortion outcomes than in Tabasco, and that may be independent of the level of restrictiveness of their abortion legislations.

The last section of the findings explores through the narratives of women their experiences with methods and providers chosen. It is interesting to observe the different outcomes when the same type of method and provider, suggesting that in addition to the type of methods and providers used, how methods are used and steps taken by providers are crucial abortion outcomes. Women who self- induced abortion, or who sought the procedure from a gynecologist in an illegal setting or from a traditional midwife, using the same methods, can come out with vastly different outcomes. In some cases, the experience was extremely bad and risky while for other women it was a relatively safe and less traumatic experience. In contrast, women who went to a safe provider in Mexico City always had a good experience with abortion. The procedure was performed safely with no complications. Similarly, women who self-induced the abortion with misoprostol with the support of an NGO knowledgeable of the use of the medication, had a safe abortion with no complications.

From this analysis, four observations can be made. First, that a high proportion of women self-induced the abortion using misoprostol suggests that use of the method has increased over time and has shaped the pattern of abortion in our study areas, and perhaps the country as a whole. Second, that Queretaro and the State of Mexico are geographically nearer to Mexico City where abortion in legal, than Tabasco resulted in women from the first two states benefiting from the liberal abortion law in Mexico City. As noted earlier, no woman from Tabasco, which is the farthest from Mexico City, travelled to the city for an abortion to take advantage of the availability of safe legal abortion. Third, women from Queretaro and the State of Mexico also benefited from telephone hotlines and NGO dedicated to harm reduction; in all of these cases, women who self induced the abortion with misoprostol and who had the help of the hotline-NGO had no complications and their abortion experiences were positive. Fourthly, the degree of legislative restrictions does not seem to be associated with the pattern of abortion methods or providers used or the outcome of the procedure. Nevertheless, legislation restriction in general (i.e. independently of the degree of restrictiveness) makes erratic the outcome of the process of abortion, in some cases, women may end up with a safe abortion and in another occasions not. Some abortion providers who advertise themselves as gynecologist were either not well trained on abortion methods recommended by WHO [22] or were not legitimate gynecologist (i.e. fake or impostors), as mentioned in the narratives of the women. Evidently, in settings with restrictive laws, women will have abortions with providers that advertise their services as gynecologist or medical personnel even if they are not medical personnel. Such providers are very common in restrictive settings since they are not accountable to a government institution because of the clandestine nature of their abortion services. The consequence is that many of those abortions end up being unsafe, some with very serious complications.

To address the challenges and problems faced by women living in the states of Mexico with restrictive abortion laws, we offer the following recommendations. Certainly, effort should continue to be made to achieve legalization of abortion in all states. This is essential for reducing unsafe, clandestine procedures and making abortion safe. A situation with liberal abortion laws and policies that make safe abortion available to all sub-groups of women is the ultimate goal that will ensure the well-being of women and their families. But achieving legalization of abortion often takes a long time.

In the meantime, we recommend that some immediate actions be taken to improve the pathway to and safety of abortion in restrictive settings in Mexico. It appears that Queretaro and the State of Mexico have a greater presence of NGOs and their harm reduction strategies have resulted in positive outcomes with respect to abortion access in those states. Furthermore, information about Mexico City laws and services would not have diffused to remote areas, as evident from the findings in Tabasco. Therefore, in the meantime and before decriminalization of the law is achieved, we recommend the following immediate actions: First, effort to promote harm reduction strategies in different states of the country, in particular those with a limited presence or none of reproductive health NGOs, and states far away from Mexico City, as our findings indicate, self-induced abortions using misoprostol with adequate information, such as from NGOs with hotline services, do result in positive experiences and safe abortions, despite the legal restrictions. Second, promotion of accurate information about self-use of misoprostol where abortion is legally restricted is recommended, this information may be provided by NGOs but also by medical personnel. This approach was successfully used in Uruguay before the legalization of abortion: doctors gave precise information on medical abortion but could not perform the abortion as it was illegal. As WHO recommended, adequate information about misoprostol should be accessible in restrictive setting, since people will use the method with or without correct information. Third, to achieve the goal of improving access to correct information about misoprostol use and prevent women from seeking unsafe abortions using dangerous methods and untrained providers, certain specific strategies can be developed and utilized by NGOs and friendly reproductive health services to reach women. Examples include use of the internet, social media and street market exhibition with information and links/referral to safe abortion services in Mexico City. Fourth, post abortion care (PAC) services should continue to be made available in public and private facilities and access should be enhanced for all categories of women. Effort should be made to ensure that all facilities allowed to provide PAC are equipped with WHO recommended methods of PAC provision and that facility staff members are adequately trained in the use of those methods and in discharging their responsibilities without judgmental attitudes towards women seeking PAC.

Findings from this study suggest the need for future research, to address gaps in the literature on abortion in Mexico. Specifically, there is need for more research to better understand the practices around use of traditional medicine, midwives and concoctions for abortion in the country. Many women in the state of Tabasco resort to these types of providers for abortion. Yet little is known about the ramifications of abortions obtained through these providers and methods, including the complications associated with them. It is of policy and program importance to learn more about how the traditional midwives practice abortion provision and the information and advice they give to women regarding preventing future unintended pregnancies and in case they develop complications due to the abortion. Further research is also needed about self-use of misoprostol for abortion by women. Several women in this study who used misoprostol do not know exactly what they were given by the provider. It will be informative to know how women who self-induced using misoprostol seek and obtain the method. For example, it will be helpful to know where they get the method from in a restrictive setting, the quality of the medication as well as the adequacy of information they received.

## Supporting information

S1 Appendix Socio-demographic and geographical characteristics of the study sites. (DOCX)

S2 Appendix Study participant’s characteristics. (DOCX)
