# Supplementary material for: Women's abortion seeking behavior under restrictive abortion laws in Mexico
Source: PLoS One. 2019 Dec 27;14(12):e0226522. doi: 10.1371/journal.pone.0226522 (PMC6934271; doi:10.1371/journal.pone.0226522)
Supplement: S1 Appendix — (DOCX) [file pone.0226522.s001.docx]

**S1 Appendix. Socio-demographic and geographical characteristics of the study sites.**

**S1 Table.** Selected demographic, economic and geographic characteristics of three states in Mexico: Queretaro, Tabasco and State of Mexico.

| Selected demographic, economic and geographical characteristics | State of Queretaro (“most restrictive” abortion law) [1] | State of Tabasco (“moderate restrictive” abortion law) [1] | State of Mexico (“least restrictive” abortion law) [1] | National |
| --- | --- | --- | --- | --- |
|  |  |  |  |  |
| Distance in hours to Mexico City in public transport [2–4] | 3 hours | 13 hours | 1 hour 20 minutes |  |
| Total fertility rate^i^ [5] | 2.1 | 2.3 | 2.0 | 2.2 |
| Contraceptive preval.^ii^ [5] | 72.4 | 69.5 | 77.8 | 72.3 |
| If last birth not desired then^iii^ [5] | 45.0 | 43.6 | 46.1 | 40.9 |
| % Never married Sexually Active^iv^ [5] | 20.1 | 10.7 | 19.0 | 16.5 |
| Abortion incidence rate p/1000 women 15-44 [6] | 43.4 | 58.9 | 54.4 | 38.0 |
| Abortion complic. rate due to abortion p/1000 women 15-44^v^ [6] | 6.4 | 8.7 | 6.7 | 5.9 |
| % Pop in poverty (earns less than two min. Salaries)^vi^ [7] | 25.9 | 37.0 | 35.3 | 37.4 |
|  |  |  |  |  |

Sources: See references [1-7].

Notes: i) own calculations, Total Fertility Rate 2011-2013; ii) own calculations, women currently in union 15-49 years, estimate for 2014; iii) own calculations, women 15-49, estimate of last birth in the past 3 years desired; iv) own calculations, women 15-49; v) Abortions estimates refer to the year 2009 and vi) Earns less than two minimum monthly salaries in 2010.

Despite differential abortion legislations and geographic differences, demographic indicators do not vary much among women across the three states. In the three states, fertility is near replacement level, contraceptive use is high, with Tabasco women reports slightly lower contraceptive use and a smaller proportion of sexually active never married, indicative that Tabasco is a more conservative society in its reproductive health behavior. Women in the three states have difficulties preventing unplanned pregnancies, nearly half of the women’s last births were not desired and abortion indicators are relatively high, with Tabasco presenting the highest level, followed by State of Mexico and then Queretaro. Tabasco is the state where the incidence of abortion and abortion complications are among the highest in the country. The proportion of the population that is poor is not negligible in the three states ranging from 26 to 37%. The poverty level of these states follows the differential patterns for the level of abortion incidence and morbidity: the poorest state is Tabasco and has the highest abortion incidence and hospital complications related to abortion, followed by the State of Mexico with lower abortion incidence and morbidity. The least poor state, Queretaro, also reports the lowest abortion incidence.

**References**

1. Grupo de Información en Reproducción Elegida (GIRE). Legislación de aborto por entidad federativa, [Internet]. 2016. Available from: https://gire.org.mx/consultations/causales-de-aborto-en-codigos-penales-estatales/?type=

2. Ciudad de Mexico-Toluca [Internet]. Autobuses Mexico. 2013 [cited 2018 Jan 8]. Available from: https://www.autobusesmexico.com/autobus/mexico-df-toluca

3. Ciudad de Mexico-Queretaro [Internet]. Autobuses Mexico. 2015 [cited 2018 Jan 8]. Available from: https://www.autobusesmexico.com/autobus/mexico-df-central-del-norte-queretaroqro

4. Mexico City-Villahermosa [Internet]. Reservamos. [cited 2018 Jan 8]. Available from: https://www.reservamos.mx/autobuses-de/ciudad-de-mexico-a-villahermosa

5. Special tabulations of data from Encuesta Nacional de la Dinámica Demográfica (ENADID 2014), Mexico.

6. Juárez F, Singh S, Maddow-Zimet I, Wulf D. Unintended pregnancy and induced abortion in Mexico: Causes and consequences (Embarazo no planeado y aborto inducido en México: Causas y consecuencias). New York. 2013;

7. Consejo Nacional de Población (CONAPO). Índice de marginación por entidad federativa y municipio [Internet]. Vol. 978. 2016 [cited 2018 Jan 8]. 2015 p. Available from: https://www.gob.mx/conapo/documentos/indice-de-marginacion-por-entidad-federativa-y-municipio-2015
