# Supplementary material for: Women's abortion seeking behavior under restrictive abortion laws in Mexico
Source: PLoS One. 2019 Dec 27;14(12):e0226522. doi: 10.1371/journal.pone.0226522 (PMC6934271; doi:10.1371/journal.pone.0226522)
Supplement: S2 Appendix — (DOCX) [file pone.0226522.s002.docx]

**S2 Appendix. Study participant’s characteristics.**

**S2 Table.** Study participants by socio-demographics and pregnancy and abortion experiences of Queretaro, Tabasco and State of Mexico.

| Number of cases | Age in yrs | Distrib. by age | Marital Status | | Educational level | | | | Induced abortion (#) | | Number of children ever born | | | |
| --- | --- | --- | --- | --- | --- | --- | --- | --- | --- | --- | --- | --- | --- | --- |
|  |  |  | Single | Married/ Union | Primary compl. or incompl.  (0-6yrs) | Secondary compl. or incompl.  (7-9yrs) | High school compl. or incompl  (9-12yrs) | Univer. compl. or incompl. (13 or more yrs) | 1 | 2 or more | 0 | 1 | 2 | 3 or more |
|  |  |  |  |  |  |  |  |  |  |  |  |  |  |  |
| 2 | 18-19 | 3.3 | 1 | 1 | 0 | 2 | 0 | 0 | 2 | 0 | 0 | 2 | 0 | 0 |
| 18 | 20-24 | 30.0 | 11 | 7 | 3 | 4 | 5 | 6 | 18 | 0 | 9 | 7 | 1 | 1 |
| 21 | 25-29 | 35.0 | 7 | 14 | 1 | 7 | 6 | 7 | 18 | 3 | 8 | 3 | 7 | 3 |
| 10 | 30-34 | 16.7 | 5 | 5 | 0 | 1 | 8 | 1 | 10 | 0 | 2 | 1 | 4 | 3 |
| 9 | 35+ | 15.0 | 6 | 3 | 0 | 2 | 6 | 1 | 7 | 2 | 0 | 1 | 4 | 4 |
|  |  |  |  |  |  |  |  |  |  |  |  |  |  |  |
| Total = 60 |  |  | 30 | 30 | 4 | 16 | 25 | 15 | 55 | 5 | 19 | 14 | 16 | 11 |
| % |  | 100.0 | 50.0 | 50.0 | 6.7 | 26.7 | 41.7 | 25.0 | 91.7 | 8.3 | 31.7 | 23.3 | 26.7 | 18.3 |

Nearly two thirds of the women in the study were 20-29 years old, the age group where women tend to have most of their births (65%). The state with the highest proportion of women in the age group 20-29 was Queretaro (85%). The corresponding proportion in the State of Mexico and Tabasco is 55%. Half of the women were single (50%), but this proportion is more pronounced for Queretaro (75%). On average two thirds of participant women had 10 or more years of schooling (66.7%): The proportion was highest in the State of Mexico (85%), 60% in Queretaro and 55% in Tabasco. Women with no children was nearly one third (31.7%) of the sample and 50% had 1-2 children. The proportion childless was 25% in the State of Mexico, 65% in Queretaro and 5% in Tabasco. In general, in the three states, most women reported having had only one abortion, the one she narrates in the interviews (91.7%).
